# Supplementary figures and images for: Pre-Emptive Use of Rituximab in Epstein–Barr Virus Reactivation: Incidence, Predictive Factors, Monitoring, and Outcomes
Source: Int J Mol Sci. 2023 Nov 7;24(22):16029. doi: 10.3390/ijms242216029 (PMC10671524; doi:10.3390/ijms242216029)

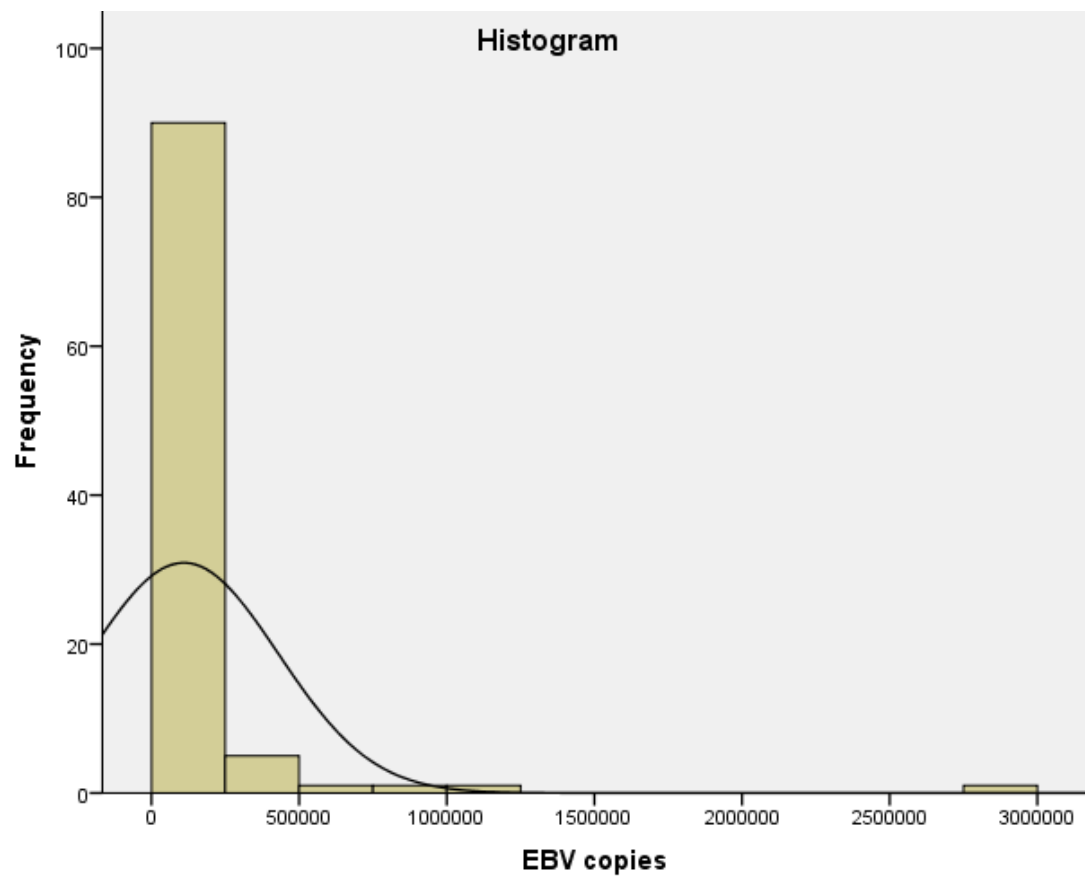

**Figure S1:** EBV copies frequency distribution plot.

Supplement: Supplementary file 1 [file ijms-24-16029-s001.zip › ijms-2624337-supplementary/Figure S1.pdf]

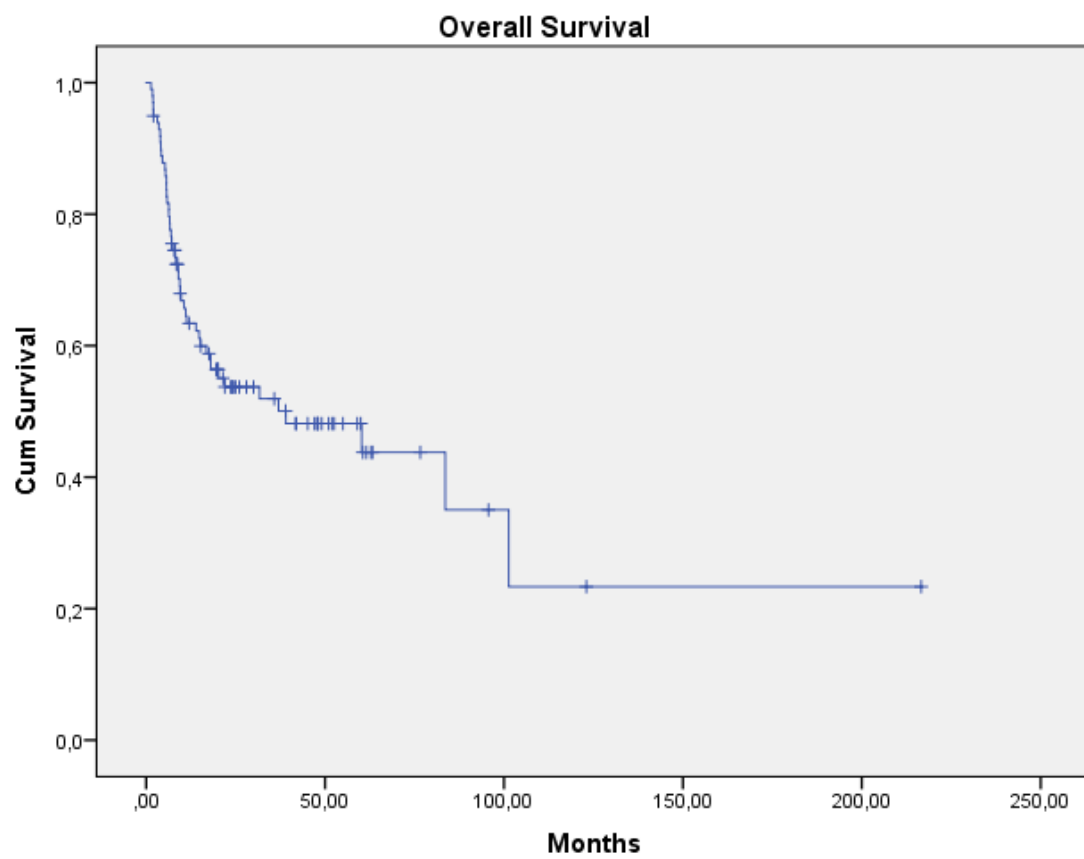

**Figure S2:** COX regression analysis.

Supplement: Supplementary file 1 [file ijms-24-16029-s001.zip › ijms-2624337-supplementary/Figure S2.pdf]

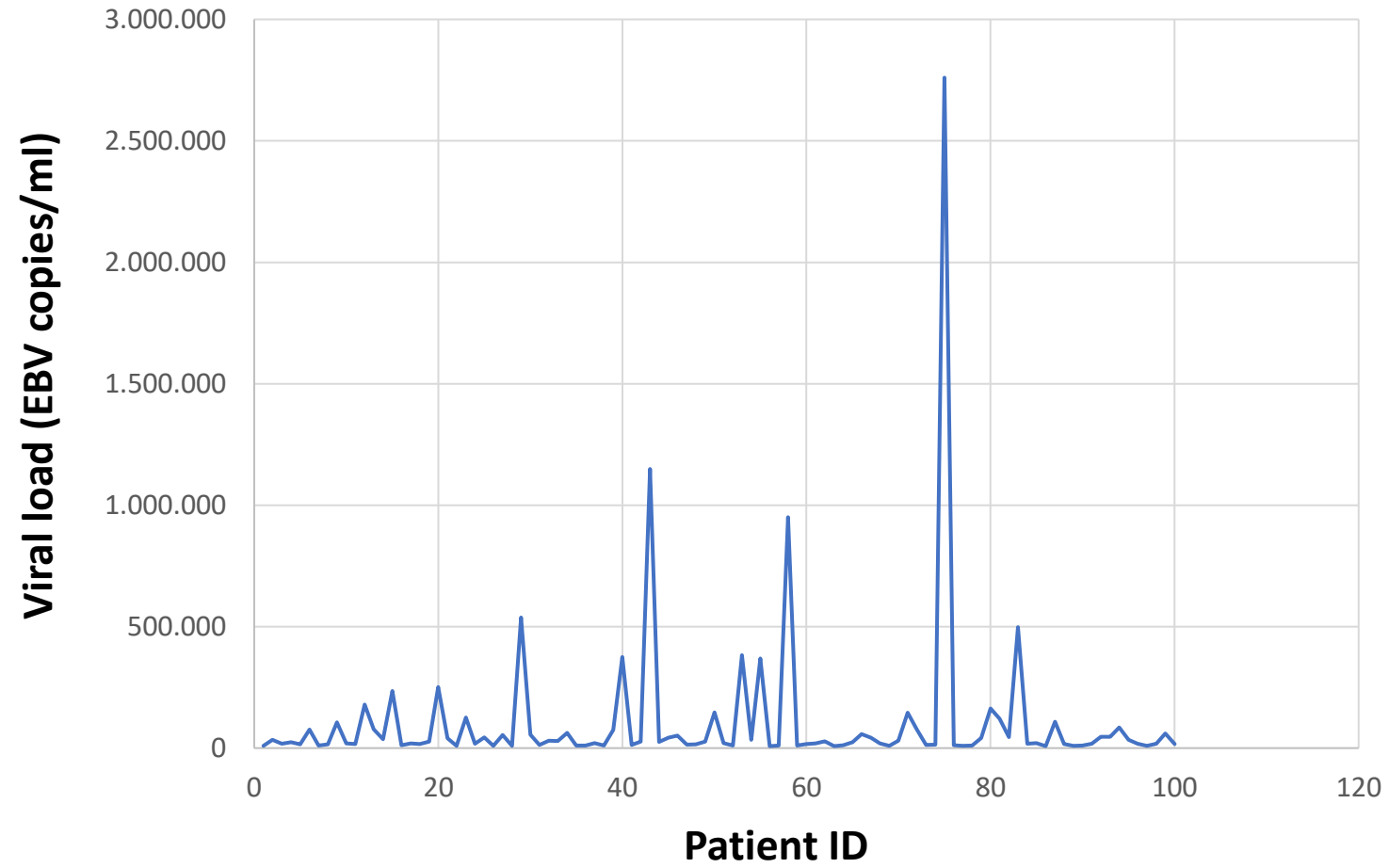

**Figure S3.** Scatter plot for EBV copies/ml before treatment per patient.

Supplement: Supplementary file 1 [file ijms-24-16029-s001.zip › ijms-2624337-supplementary/Figure S3.pdf]

**Supplemental Figure S4.** Kaplan-Meier curve for relapse-free (RFS, A) and overall survival (OS, B).

A.

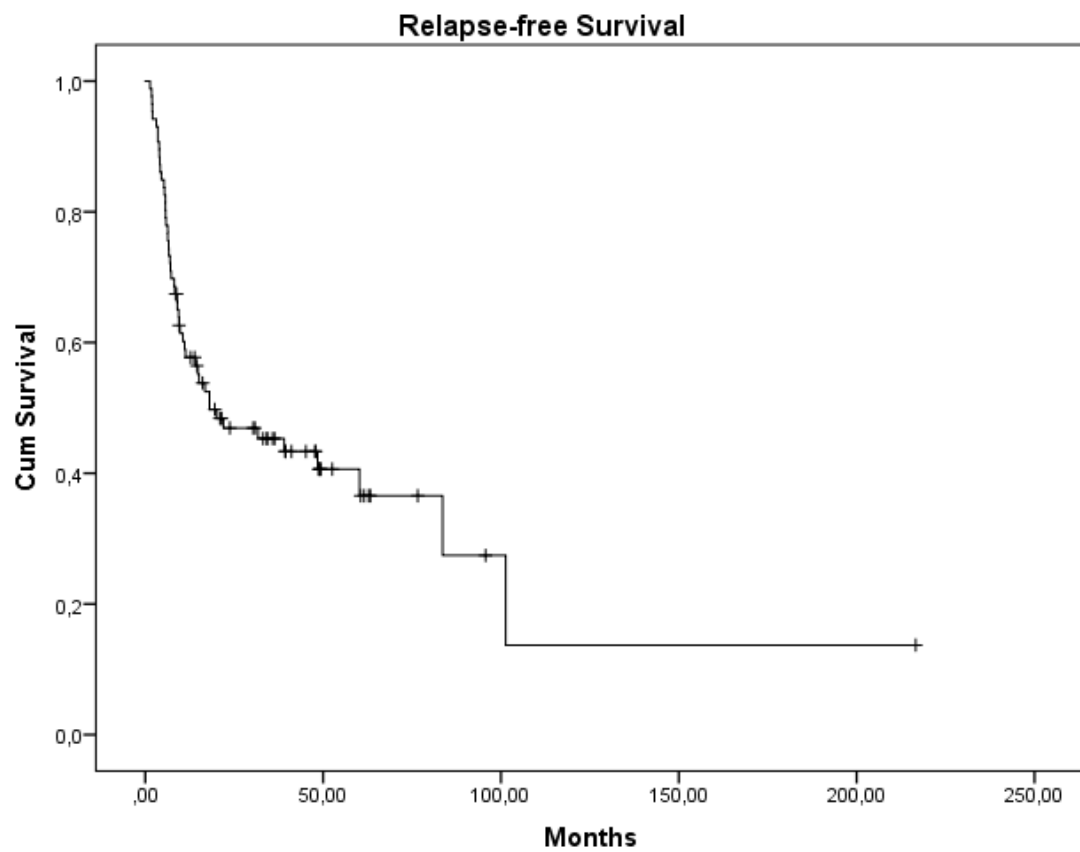

B.

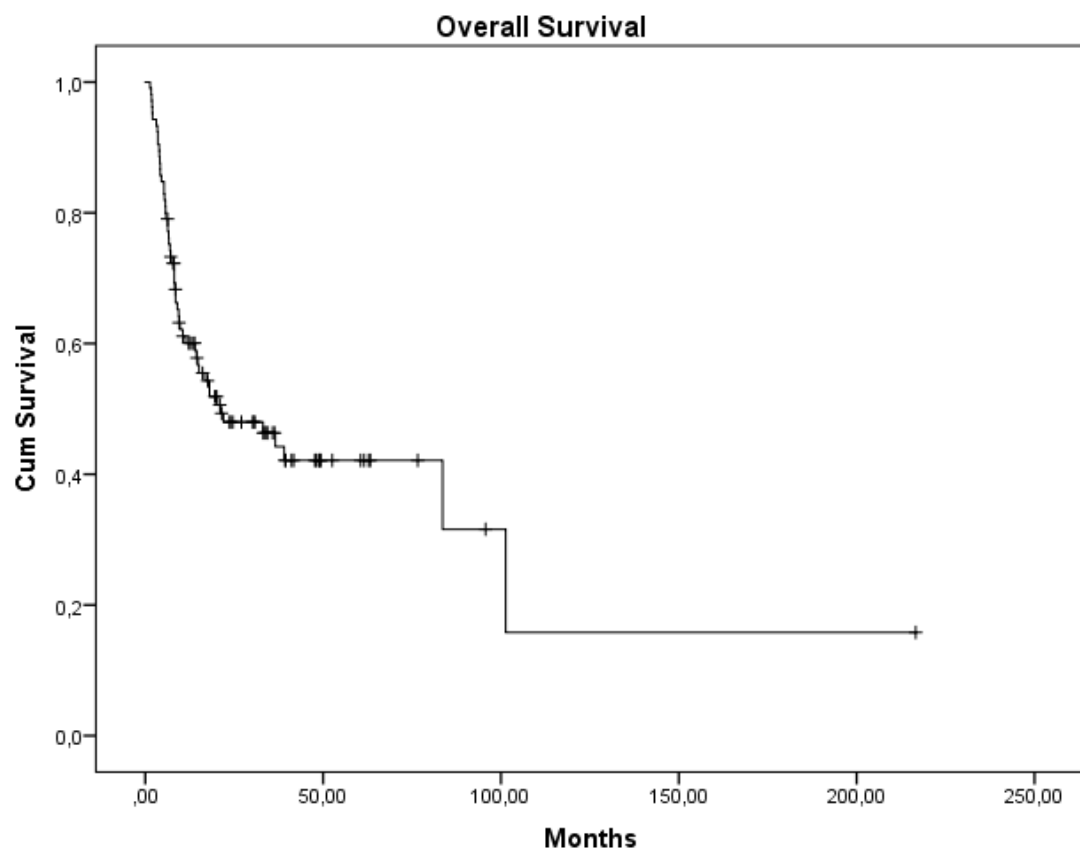

Supplement: Supplementary file 1 [file ijms-24-16029-s001.zip › ijms-2624337-supplementary/Figure S4.pdf]

**Supplemental Figure S5.** Cumulative incidence of treatment-related mortality

(TRM) in months.

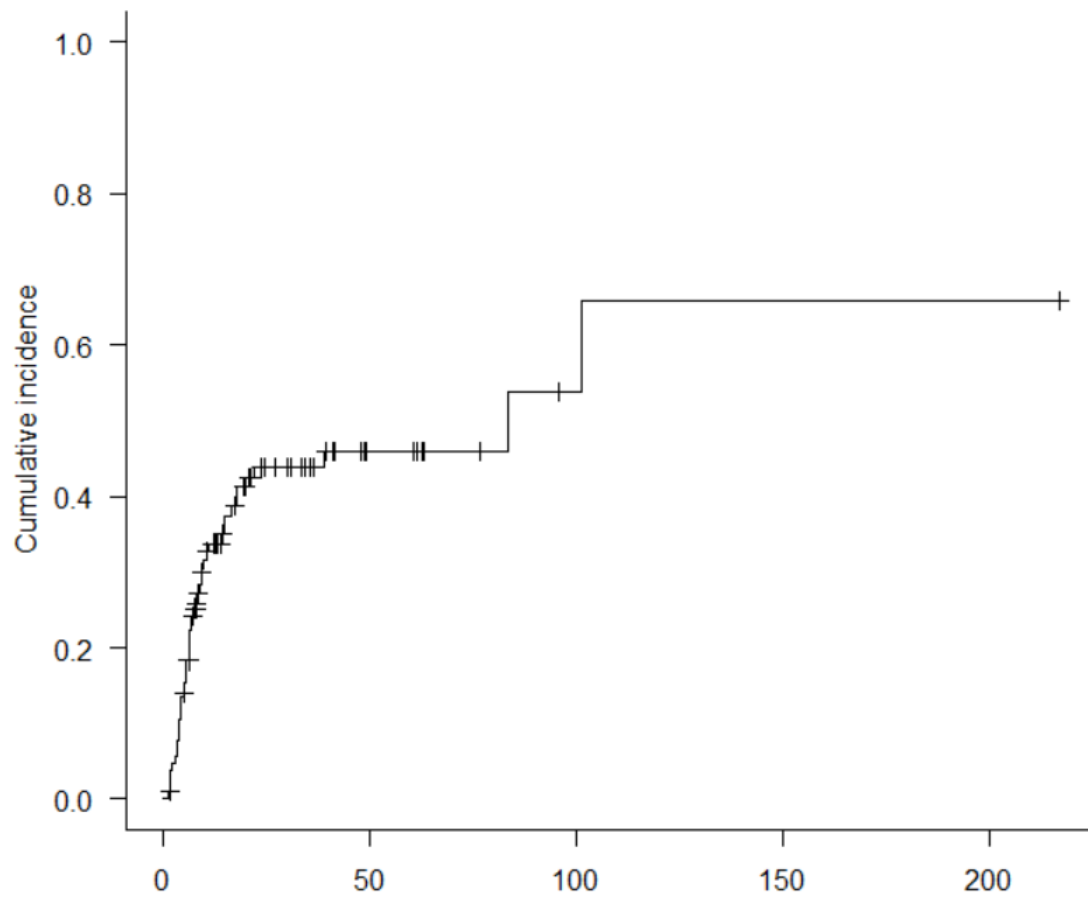

Supplement: Supplementary file 1 [file ijms-24-16029-s001.zip › ijms-2624337-supplementary/Figure S5.pdf]
